# Areas of Interest and Attitudes towards the Pharmacological Treatment of Attention Deficit Hyperactivity Disorder: Thematic and Quantitative Analysis Using Twitter

**DOI:** 10.3390/jcm10122668

**Published:** 2021-06-17

**Authors:** Miguel Angel Alvarez-Mon, Laura de Anta, Maria Llavero-Valero, Guillermo Lahera, Miguel A. Ortega, Cesar Soutullo, Javier Quintero, Angel Asunsolo del Barco, Melchor Alvarez-Mon

**Affiliations:** 1 Service of Psychiatry and Mental Health, Hospital Universitario Infanta Leonor, 28031 Madrid, Spain; lanta.79@gmail.com (L.d.A.); fjquinterog@salud.madrid.org (J.Q.); 2 Department of Medicine and Medical Specialities, University of Alcala, 28805 Alcala de Henares, Spain; guillermo.lahera@gmail.com (G.L.); miguel.angel.ortega92@gmail.com (M.A.O.); mademons@gmail.com (M.A.-M.); 3 Department of Endocrinology and Clinical Nutrition, Hospital Universitario Infanta Leonor, 28031 Madrid, Spain; maria.llavero@salud.madrid.org; 4 Department of Psychiatry, University Hospital “Principe de Asturias”, 28805 Alcala de Henares, Spain; 5 CIBERSAM (Biomedical Research Networking Centre in Mental Health), 22807 Madrid, Spain; 6 Institute Ramon y Cajal for Health Research (IRYCIS), 28034 Madrid, Spain; angel.asunsolo@uah.es; 7 Louis A Faillace Department of Psychiatry and Behavioral Science, The University of Texas Health Science Centre at Houston, Houston, TX 77054, USA; Cesar.A.Soutullo@uth.tmc.edu; 8 Department of Legal Medicine, Psychiatry and Pathology, Complutense University, 28040 Madrid, Spain; 9 Department of Surgery, Medical and Social Sciences, Faculty of Medicine and Health Sciences, University of Alcalá, 28805 Alcala de Henares, Spain; 10 Biomedical Institute for Liver and Gut Diseases (CIBEREHD), Instituto de Salud Carlos III, Av. Monforte de Lemos, 3-5, 28029 Madrid, Spain; 11 Service of Internal Medicine and Rheumatology, Autoimmune Diseases University Hospital “Principe de Asturias”, 28805 Alcala de Henares, Spain

**Keywords:** ADHD, social media, Twitter, pharmacotherapy, stimulants, alpha-2-adrenergic agonists, non-stimulants

## Abstract

We focused on tweets containing hashtags related to ADHD pharmacotherapy between 20 September and 31 October 2019. Tweets were classified as to whether they described medical issues or not. Tweets with medical content were classified according to the topic they referred to: side effects, efficacy, or adherence. Furthermore, we classified any links included within a tweet as either scientific or non-scientific. We created a dataset of 6568 tweets: 4949 (75.4%) related to stimulants, 605 (9.2%) to non-stimulants and 1014 (15.4%) to alpha-2 agonists. Next, we manually analyzed 1810 tweets. In the end, 481 (48%) of the tweets in the stimulant group, 218 (71.9%) in the non-stimulant group and 162 (31.9%) in the alpha agonist group were considered classifiable. Stimulants accumulated the majority of tweets. Notably, the content that generated the highest frequency of tweets was that related to treatment efficacy, with alpha-2 agonist-related tweets accumulating the highest proportion of positive consideration. We found the highest percentages of tweets with scientific links in those posts related to alpha-2 agonists. Stimulant-related tweets obtained the highest proportion of likes and were the most disseminated within the Twitter community. Understanding the public view of these medications is necessary to design promotional strategies aimed at the appropriate population.

## 1. Introduction

Attention deficit hyperactivity disorder (ADHD) is one of the most common neuropsychiatric disorders of childhood and adolescence, often persisting into adulthood [1]. The reported prevalence of ADHD in children varies from 2 to 18 percent [2,3]. ADHD is associated with negative health outcomes and marked impairment in academic, occupational and social functioning [4,5].

The treatment of ADHD is complex and may involve behavioral, psychological and educational interventions, as well as medication [6]. Different pharmacological treatments have shown efficacy in reducing ADHD symptoms and improving daily functioning [6]. As has been reported, however, the efficacy of these treatments is not homogenous, nor is the frequency and pattern of associated side effects [6]. The choice of the initial medication depends upon a number of factors, including the individual preferences of the clinician, patient and family [6]. Furthermore, adherence to the treatment regimen is critical for the efficacy of the medical intervention [7,8]. Determinants of patient behavior, including adherence to medication and one’s own lifestyle habits, are influenced by patients’ experiences, attitudes and opinions with regard to their treatment [7,8]. In order to better optimize medical treatments for the management of ADHD, analyses of the opinions of patients and their families are therefore required.

Social media platforms are increasingly being leveraged by researchers for public health surveillance, intervention delivery, the study of attitudes toward health behaviors and diseases, predictions on diseases, and insight into the medical experiences of patients [9,10,11,12]. In particular, Twitter is the most commonly used social media platform within health research, and content analysis is the most common approach [13,14]. In this context, the exploration of tweets discussing perceptions of medications for better understanding, compliance and therapeutic decision making has been sufficiently established [15,16].

Moreover, research on patients’ beliefs and attitudes has traditionally relied on surveys, interviews and clinical trials [17,18]. However, social media may also allow for a wider range of patients’ voices to be heard, including those perspectives from patients reluctant to participate in surveys or research. In addition, since social media posts are spontaneous in nature, they may be more reflective of what patients truly experience than surveys conducted by researchers, which rely on structured, formal interviews [19,20,21]. Moreover, postings can be collected nearly in real time, thereby avoiding recall bias. Consequently, platforms such as Twitter may provide a useful insight into patients’ beliefs.

Finally, the analysis of tweets on psychiatric disorders is a recently significant area of study for understanding the sentiments of society, patients and health professionals [22,23,24]. That being said, topics of medical and non-medical interest among Twitter users with relation to ADHD treatment have not yet been established, with the dissemination of ADHD-related tweets remaining unknown.

In this study, we have hypothesized that, firstly, the pharmacological treatment for ADHD is an area of interest for Twitter users and that, secondly, a diverse perception towards the different drug treatments available can be observed. More specifically, the aims of this multidisciplinary research were to investigate the interest and social considerations of Twitter users towards approved pharmacological treatments for ADHD. In addition, we investigated the dissemination of these tweets.

## 2. Materials and Methods

### 2.1. Data Collection

In this observational quantitative and qualitative study, we focused on searching for tweets that referred to medications approved for the treatment of ADHD: Adderall, Dexedrina, Dextrostat, Focalin, Metilin, Ritalin, Metadate CD (methylphenidate), Ritalin LA (methylphenidate), Adderal-XR, Vyvanse (Lisdexamfetamine), Concerta, Daytrana, Focalin XR, Quillivant XR (methylphenidate), Intuniv (guanfacine), Kapvay (clonidine) and Strattera (Atomoxetine). The inclusion criteria for tweets were: (1) being posted publicly; (2) using any of the previously mentioned hashtags; (3) being posted between 20 September and 31 October 2019; (4) being written in English or Spanish. The six-week period was chosen to avoid any potential bias in the content of the tweets. We collected the number of likes each tweet generated, the date and time of each tweet, a permanent link to the tweet and each user’s profile description. In addition, we obtained a list of the ten hashtags most frequently associated with the hashtags of our study.

### 2.2. Search Tool

We used the Twitter Firehose data stream, which is managed by Gnip and allows access to 100% of all public tweets that match a certain criteria (query). In our study, the search criteria were the previously mentioned hashtags.

### 2.3. Content Analysis Process

**Figure 1:**
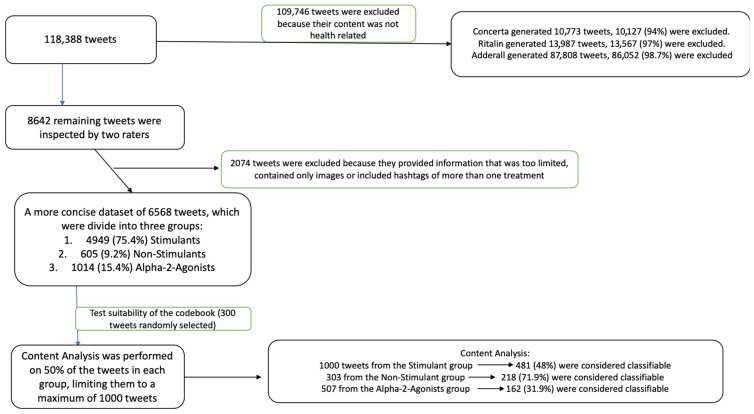
Flowchart of data management.

All 118,388 retrieved tweets were included in the dataset (Figure 1). First, we excluded those tweets mentioning any of the aforementioned medications in an unrelated context. For example, Concerta is also the name of a political party in Chile. In this case, any tweets referring to the political party were omitted. Secondly, we excluded all tweets, including hashtags and keywords, not related to health (e.g., political issues). Specifically, Concerta and Ritalin generated 10,773 and 13,987 tweets, respectively, but 10,127 (94%) and 13,567 (97%), respectively, were not related to health. Indeed, most of them included hashtags (#mesacentral, #apoyofirmado, #tumbamadre, #lamarchamasgrande, #Pinerarenuncia) or keywords related to political conflict occurring in Chile. Similarly, Adderall generated 87,808 tweets, of which 86,052 (98.7%) included hashtags or keywords related to political issues (e.g., Trump, impeachment).

All 8642 remaining tweets were inspected by two raters (M.A.A.-M. and L.d.A.). First, we scanned all of the tweets and excluded 2074 that provided information that was too limited, contained only images or included hashtags of more than one treatment. This process led to the creation of a more concise dataset of 6568 tweets, which we divided into three groups: 4949 (75.4%) stimulants, 605 (9.2%) non-stimulants and 1014 (15.4%) alpha-2-adrenergic agonists.

Next, we created a codebook based on our research questions, our previous experience in analyzing tweets and what we determined to be the most common themes. M.A.A.-M. and L.d.A. analyzed 300 tweets separately to test the suitability of the codebook. Discrepancies were discussed between the raters and with another author (M.L.-V.). After revising the codebook, the raters then proceeded to perform a content analysis of 50% of the tweets in each group, limiting them to a maximum of 1000 tweets randomly selected. Thus, we manually analyzed 1000 tweets from the stimulant group, 303 from the non-stimulant group and 507 from the alpha-2 agonist group (Figure 1). Classification criteria and examples of tweets are shown in Table 1.

### 2.4. Measuring Influence and Interest on Twitter

We analyzed the number of likes generated by each tweet as an indicator of user interest on a given topic. We also measured the potential reach and impact of all analyzed hashtags. Impact is defined as a numerical value representing the potential views a tweet may receive, while reach is defined as a numerical value measuring the potential audience of the hashtag.

In addition, we measured how positive or negative a hashtag was on a scale from 1 (negative) to 100 (positive). Sentiment analysis tools analyze all words contained in a tweet, and each word has its own score that can vary depending on the context. The average score of all the tweets with a certain hashtag determines that hashtag’s overall sentiment score.

### 2.5. Ethical Considerations

This study was approved by the Research Ethics Committee of the University of Alcala (OE 14_2020).

### 2.6. Statistical Analysis

A descriptive study of the sample was performed, describing the variables by their absolute and relative frequencies. The percentages found were compared using the chi-square test. In the case of quantitative variables, it was checked whether they followed a normal distribution using the Kolmogorof–Smirnof test. As this was not the case, non-parametric tests were used. The Kruskal–Wallis test was used for comparisons of median values among three groups, followed by post hoc testing using a Bonferroni-adjusted alpha level.

## 3. Results

### 3.1. Stimulants Accumulated the Most Interest among Twitter Users

According to the codebook, 521 (52%) of the stimulant tweets, 85 (28.1%) of the non-stimulant tweets and 345 (68.1%) of the alpha-2 agonist tweets were considered unclassifiable. These tweets shared information or news either about the commercialization of the medication, business-related information, or mentions of treatments for other disorders apart from ADHD. In the end, 481 (48%) of the tweets in the stimulant group, 218 (71.9%) in the non-stimulant group and 162 (31.9%) in the alpha agonist group were considered classifiable (Figure 1). In terms of the content of these tweets, the mention of the specific medications was related to their efficacy, side effects or adherence to treatment for ADHD (Table 1). Moreover, these coding categories were not mutually exclusive in the sense that a generated tweet could be listed under more than one category.

There were significant differences in the percentage of tweets with medical efficacy content between the three groups of drugs (Table 2). The percentage of tweets related to the efficacy of the alpha-2 agonist group was higher than that found in the stimulant and non-stimulant groups. Furthermore, the alpha-2 agonist group also had the highest percentage of tweets containing a positive description of the efficacy of their use (74.1%). Similar results were observed in the percentage of tweets addressing efficacy, as well as the valuation of that efficacy among the stimulant and non-stimulant groups.

The analysis of the content related to the side effects of the treatments also showed significant differences between the three groups of drugs (Table 2). The alpha-2 agonist group had the highest percentage of tweets with content related to side effects and accumulated the highest percentage of those tweets with a negative valuation (72.8%). In contrast, the stimulant group had the lowest percentage of negative valuations towards side effects (49.1%). There were not any significant differences in the percentages of those tweets mentioning treatment adherence between the three groups of drugs, being that they were all low.

### 3.2. Scientific Links Were Mainly Found in Alpha-2 Agonist-Related Tweets

We investigated the use of sources defined by the inclusion of links within the tweet. The links were categorized as scientific or non-scientific sources. Of the tweets related to ADHD, 185 out of the 861 (21.5%) included a reference source, the majority of which were scientific in nature (94.6%). We found significant differences between the percentages of tweets containing a reference link between the three groups of drugs (*p* < 0.001) (Table 2). Those tweets related to alpha-2 agonists had the highest percentage of links, of which most were scientific. In contrast, tweets related to the stimulant drug group had the lowest percentage of links (3.1%).

We observed that the percentages of tweets with negative or positive content related to the efficacy of treatments were different among those tweets both including and not including a link (*p* < 0.001) (Table 3). The negative opinion of treatment efficacy was higher in those without a link (8.1%). In contrast, the percentage of tweets related to side effects was higher among those with a link than in those without one included (*p* < 0.001). Interestingly, the use of links in tweets with adherence content was very low (0.5%).

We studied the use of links in the three groups of treatments. We found a different pattern of distribution of links within the different categories. Within the group of alpha-2 agonist tweets, we observed that the majority of the tweets with a link were focused on efficacy and side effects (Table 4). In contrast, within the non-stimulant group, references to efficacy were mainly posted without a link. Lastly, within the stimulant drug group, efficacy was mainly addressed using a link, whereas side effects were mainly addressed without one.

### 3.3. Stimulant Related Tweets Were the Most Disseminated within the Twitter Community

We found that the probabilities of a tweet being liked among the three groups were significantly different (*p* < 0.001). Alpha-2 agonists showed a statistically significantly lower number of likes than both non-stimulant (*p* = 0.024) and stimulant (*p* < 0.001). Stimulant-related tweets accumulated the highest median of likes per tweet. In addition, we analyzed the number of likes received per tweet as classified by the inclusion or absence of a link. We found that tweets not including a link had a significantly higher median of likes per tweet than those tweets including a link (*p* = 0.041).

**Figure 2:**
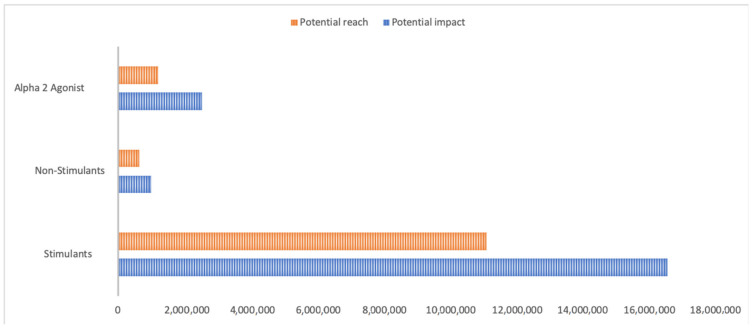
Potential reach and potential impact.

**Figure 3:**
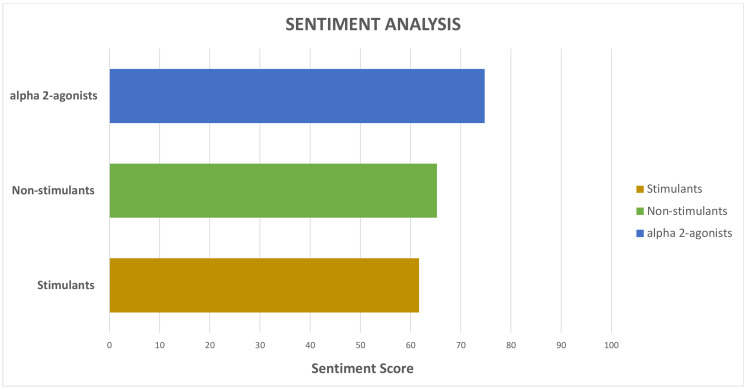
Sentiment score comparisons between alpha-2 agonists, non-stimulants and stimulants. Sentiment analysis is classifying the polarity of the tweet on a scale of 0 (very negative) to 100 (very positive).

Furthermore, we found that stimulant-related tweets had the highest potential reach and impact (Figure 2). Both parameters were markedly lower for non-stimulant and alpha-2 agonist-related tweets. Regarding the sentiment analyses of the content of the tweets, we found that it was positive for all three groups (Figure 3).

## 4. Discussion

### 4.1. Principal Findings

In this study, we have found that Twitter users show a great interest in ADHD drugs, mainly focusing on stimulants. These tweets are centered on the efficacy and side effects of ADHD treatment. Tweets containing a positive consideration of efficacy were mainly observed in those posts related to alpha-2 agonists. The frequency of tweets with content related to adherence to treatment was marginal. The highest percentages of tweets with scientific links were observed in those related to alpha-2 agonists. Furthermore, those tweets referencing stimulants obtained the highest potential reach and impact.

The treatment of ADHD is complex, involving both the use of non-pharmacological tools and the prescription of drugs [6]. Regarding pharmacological treatments, different variables condition their clinical results. For instance, the efficacy of a drug for controlling disease symptoms and the frequency and intensity of side effects are considered to be critical for a treatment’s success [6]. Nevertheless, the subjective experience of a drug being used by a patient is pivotal too in terms of adherence to treatment [7,8]. Furthermore, a patient’s experience when consuming a drug is conditioned by any information or social valuations received [25]. Thus, the study of patients’ experiences with regard to the efficacy and side effects of, as well as adherence to, ADHD treatments is an area of intense focus, having been previously assessed mainly through the use of qualitative studies such as surveys and interviews [26,27]. However, contradictory results have also been reported on the different drugs employed in ADHD treatment [28].

Currently, Twitter serves as one of the predominant social platforms for disseminating perspectives publicly, giving anonymity to user testimonies and encouraging communication by people with real or perceived personal and social restrictions [29]. This anonymity also prevents the potential stigmatization of a Twitter user for his/her attitudes towards a disease, or for any physical or mental conditions they choose to disclose [30]. Thus, Twitter has become a relevant tool for the dissemination of medical information and an interesting resource for the study of individual experiences and opinions [31]. Furthermore, it has been shown that young people tend to hide information from their doctors, especially that information related to behaviors of which health care providers do not usually approve [32]. As a result, Twitter gives them the opportunity to express their experiences anonymously [33].

In this study, we have demonstrated that the use of Twitter for sharing information on patient experiences regarding ADHD treatment is significant, with tweets of this type maintaining a high frequency among those containing content more generally related to medical treatment. Nevertheless, Twitter users tend to be younger than the population at large; likewise, ADHD tends to affect a mostly younger demographic [1]. Moreover, the majority of the tweets with medical content on ADHD drug treatments were related to the stimulant group. Interestingly, these data uphold the elevated frequency of the use of these drugs in the treatment of ADHD patients globally [34].

The high frequency of tweets with content related to the efficacy and side effects of pharmacological treatments supports their significance to ADHD patients. Several studies have also examined the efficacy of each treatment on ADHD symptoms; however, with contradictory results [28]. Various reasons might explain such a discrepancy, yet this strategy for obtaining patient information is critical regardless. Additionally, it has been proposed that infodemiology may overcome the Hawthorne Effect as well as any memory recall biases common to cross-sectional surveys and questionnaire-based studies [19,35]. In terms of medical efficacy, our findings show that the alpha-2 agonist group of drugs accumulated the highest frequency of tweets with a positive valuation, even though frequency levels observed for both stimulants and non-stimulants ranked similarly. However, the alpha-2 agonist group of drugs received the highest frequency of tweets related to their side effects; interestingly, stimulants received the lowest frequency of tweets with regard to tolerability. It has been previously shown that some of the side effects of stimulant drugs have even been considered positive and actively pursued by patients [36]. These results might support the designation of stimulant drugs as the first pharmacological option for treating ADHD, as evidenced by several guidelines [6,37].

Our data also show that adherence to a pharmacological treatment is not a relevant consideration for ADHD patients who are Twitter users. Additional to this point is the fact that a similarly low frequency of tweets related to treatment adherence was found within all three groups of drugs, with a positive valuation towards adherence uncommon but slightly higher in the non-stimulant and alpha-2 agonist groups. Furthermore, the limited interest for treatment adherence found among Twitter users confirms previous studies carried out that employed other strategies [38,39].

Correct medical information is considered to be a cornerstone for the understanding of disease and subsequent patient treatments [40,41]. Currently, access to medical information has been generalized across the internet. For instance, we have found that one fifth of the content related to medical treatment included a link, a majority of which was deemed scientific in nature. This low frequency of the inclusion of links in tweets related to ADHD pharmacological treatment contrasted with those found in a study on statins [19]. Moreover, tweets including a link were twenty times more frequent in those posts referring to alpha-2 agonists than in those related to stimulants.

Different patterns in the use of links were also found between the different groups of drugs analyzed. Within the group of alpha-2 agonist tweets, for instance, the majority of tweets with a link were focused on efficacy and side effects. In contrast, among the non-stimulant group, the majority of tweets mentioning efficacy did not include a link. These results indicate the significant relevance of scientific information and medical research for ADHD patients who are Twitter users. As an example, most alpha-2 agonist medications have been approved for ADHD treatment over the last ten years, while stimulants have been used for decades. This finding therefore supports Twitter’s value as a means of communicating scientific content. However, it is worrying that only a limited number of tweets referring to ADHD treatment adherence included a scientific link, especially considering that adherence is pivotal to treatment success [7,8]. That being said, trends in information exchanged over Twitter may still be important as studies have identified that certain health behaviors can be affected [42,43].

Our study also shows that the names of those drugs used for ADHD treatment coincided with tweets referencing political, social and other non-medical content. Furthermore, we observed pejorative uses of these names by Twitter users. These findings suggest that social stigmatization towards mental health, as previously described, still persists, producing deleterious effects in the lives of people suffering from mental health conditions [44,45]. As well, the non-medical use of psychostimulant drugs, which has not always been uncovered via traditional surveys, has nevertheless been revealed through the analysis of Twitter content [46].

Clinicians themselves should therefore take into consideration information posted over social media with regard to pharmacological treatment that otherwise may not be spontaneously reported during a patient interview. This is particularly important for medications commonly abused or consumed over the counter, behaviors commonly hidden by patients from doctors [47]. In this context, social media may be deemed a friendlier place to discuss the effects of medications, especially those usually rejected by doctors. As relates to this study, an increase in the dissemination of scientific information on ADHD treatment and, in particular, the importance of the adherence to said treatment appears to be a primary objective for the medical community at large.

### 4.2. Limitations

First, Twitter may not be reflective of the general population. Secondly, researchers cannot directly measure clinical outcomes from tweets. Third, the codebook design and text analysis imply a degree of subjectivity. However, this methodology is consistent with previous medical research studies using Twitter. Furthermore, to address this last issue, our study comprised a series of countermeasures including an initial review, design of the codebook, and an agreement between coders. Although computerized machine learning methods have been tested to automatically identify and classify topics in medical research over social media, we used an analytical strategy based on raters’ clinical expertise in psychiatry, which constituted a qualitative advantage compared to other automated strategies [48]. Finally, we did not determine whether the date of FDA approval affected Twitter activity differently when comparing more recent medication to older medication.

## 5. Conclusions

This study identified interesting beliefs and opinions regarding the pharmacological treatment of ADHD that may affect patient behavior. Moreover, social media may be useful for investigating the public’s prevailing attitudes when investigating particular medications, as well as when patients report on adverse events and efficacy since both issues can affect their choice of and adherence to treatment. Public perceptions about medications could in turn help inform clinicians, particularly when developing treatment guidelines. Specific to ADHD, public opinions elucidated by this study could be used to help update guidelines, improve communication between health care professionals and patients and ultimately help to build more viable bridges between both parties.

## Figures and Tables

**Table 1:** Category, definitions and examples of classification. Usernames and personal names were removed.

Category	Examples of Tweets
**EFFICACY** (refers to the ability or inability of a treatment to provide a beneficial effect)	I like to talk about meds. been on ADHD meds almost consistently from 8 yrs old to now (19). im on Concerta right now and it’s working wonders for me. people are very strung out about medicating kids and i get that but, i really needed it as a kid and need it more as an adult. Unfortunately concerta didn’t work for me: (but everybody’s different! it was so long ago i can’t remember what my problem with it was haha...i think it just wasn’t strong enough for me. but it’s not a bad drug i know some ppl that take it! make sure you’re eating ok?
**SIDE EFFECTS** (refers to any effect that is secondary to the one intended either adverse or beneficial; we also included tweets discussing tolerability of the drug)	I just vividly remember going off Concerta as a kid because it killed my appetite and I have a hard enough time keeping weight on as it is (this 3-week depressive low has already ate away [haha] at 2 or 3 lbs) and I’m afraid of that happening again Concerta makes me want to eat three leaves of lettuce a day and id still feel bloated. I was diagnosed at an early age and was put on a few medications. I had some medications that made me very emotional. The last medication I remember being on was concerta and it flattened my mood waaay too much I was really thirsty, not eating, and super paranoid when I tried vyvanse. I remember taking my first concerta, it was the 18 mg and I was in matric. Stayed up all night like a maniac
**ADHERENCE** (refers to the degree of conformity to the recommendations about the treatment with respect to the timing, dosage or frequency)	I love the way you explained it! I was diagnosed in 2012, five years into my serving in the military. I took concerta for the first 3 months after my diagnosis and then stopped. I struggled with accepting this diagnosis. Now in 2019 I’ve accepted it & want to get help. Uhm I’m not doing this willingly. I’m all for medicine. I’m not taking my meds rn because I need to do a new examination of my diagnosis and I need to have 0 trace of concerta in my body by then. I legit can’t wait to get to take meds again. I have 72 mg Concerta, ive been on it for years but i dont take it every single day.

**Table 2:** Descriptive characteristics of the tweets considered classifiable in the content analysis, categorized by total amount per drug and category.

*N*		ALPHA-2 AGONIST	NON-STIMULANT	STIMULANT	*p*-Value
		162	218	481	
EFFICACY	No Mention	36 (22.2%)	84 (38.5%)	176 (36.6%)	
	Positive	120 (74.1%)	118 (54.1%)	270 (56.1%)	
	Negative	6 (3.7%)	16 (7.3%)	35 (7.3%)	
					*p* < 0.001
SIDE EFFECTS	No Mention	40 (24.7%)	77 (35.3%)	239 (49.7%)	
	Positive	4 (2.5%)	3 (1.4%)	6 (1.2%)	
	Negative	118 (72.8%)	138 (63.3%)	236 (49.1%)	
					*p* < 0.001
ADHERENCE	No Mention	148 (91.4%)	196 (89.9%)	451 (93.8%)	
	Positive	8 (4.9%)	12 (5.5%)	9 (1.9%)	
	Negative	6 (3.7%)	10 (4.6%)	21 (4.4%)	
					*p* = 0.1
LINK	No Mention	65 (40.1%)	145 (66.5%)	466 (96.9%)	
	Scientific	94 (58.0%)	68 (31.2%)	13 (2.7%)	
	Non-Scientific	3 (1.9%)	5 (2.3%)	2 (0.4%)	
					*p* < 0.001

For each category, total number of tweets (*n*) and relative proportions (%) are provided. Chi-square tests were conducted to assess for statistical differences.

**Table 3:** Descriptive characteristics of the tweets considered classifiable in the content analysis, categorized by either including or not including a link.

Total		WITHOUT LINK	WITH LINK	*p*-Value
		676	185	
**EFFICACY**	No Mention	238 (35.4%)	57 (30.8%)	
	Positive	382 (56.5%)	126 (68.1%)	
	Negative	55 (8.1%)	2 (1.1%)	
				*p* < 0.001
**SIDE EFFECTS**	No Mention	320 (47.3%)	36 (19.5%)	
	Positive	10 (1.5%)	3 (1.6%)	
	Negative	346 (51.2%)	146 (78.9%)	
				*p* < 0.001
**ADHERENCE**	No Mention	611 (90.4%)	184 (99.5%)	
	Positive	29 (4.3%)	0	
	Negative	36 (5.3%)	1 (0.5%)	
				*p* < 0.001

For each category, total number of tweets (n) and relative proportions (%) are provided. Chi-square tests were conducted to assess for statistical differences.

**Table 4:** Use of links in the different content categories of the tweets related to the three different groups of pharmacological treatments.

		ALPHA-2 AGONIST		NON-STIMULANTS		STIMULANTS	
		WITHOUT LINK	WITH LINK	WITHOUT LINK	WITH LINK	WITHOUT LINK	WITH LINK
**Total**		65	97	145	73	466	15
**SIDE EFFECTS**	NM	26 (40%)	14 (14%)	66 (45.5%)	11 (15.1%)	288 (48.9%)	11 (73.3%)
	+	1 (2%)	3 (3%)	3 (2.1%)	0	6 (1.3%)	0
	−	38 (58%)	80 (82%)	76 (52.4%)	62 (84.9%)	232 (49.8%)	4 (26.7%)
			*p* = 0.001		*p* < 0.001		*p* = 0.17
**EFFICACY**	NM	24 (37%)	12 (12%)	42 (29%)	42 (57.5%)	173 (37.1%)	3 (20%)
	+	36 (55%)	84 (87%)	87 (60%)	31 (42.5%)	259 (55.6%)	11 (73.3%)
	−	5 (8%)	1 (1%)	16 (11%)	0	34 (7.3%)	1 (6.7%)
			*p* < 0.001		*p* < 0.001		*p* = 0.37
**ADHERENCE**	NM	52 (80%)	96 (99%)	123 (84.8%)	73 (100%)	436 (93.6%)	15 (100%)
	+	8 (12%)	0	12 (8.3%)	0	9 (1.9%)	0
	−	5 (8%)	1 (1%)	10 (6.9%)	0	21 (4.5%)	0
			*p* < 0.001		*p* = 0.002		*p* = 0.60

Percentages (%) were calculated with respect to the total number of tweets generated without or with links in each group of treatments and content category. NM = no mention. + = positive. − = negative.

## Data Availability

The data that support the findings of this study are available from the corresponding author upon reasonable request.
